# Towards a more transparent HTA process in Poland: new Polish HTA methodological guidelines

**DOI:** 10.1080/20016689.2017.1355202

**Published:** 2017-07-26

**Authors:** Krzysztof Lach, Michal Dziwisz, Cécile Rémuzat, Mondher Toumi

**Affiliations:** ^a^ Department of Pricing & Reimbursement and Market Access, Creativ-Ceutical, Cracow, Poland; ^b^ Department of Pricing & Reimbursement and Market Access, Creativ-Ceutical, Paris, France; ^c^ Laboratoire de Santé Publique, Aix-Marseille Université, Université de la Méditerranée, Marseille, France

**Keywords:** Health Technology Assessment, guideline, methods, Poland

## Abstract

**Introduction**: Health technology assessment (HTA) in Poland supports reimbursement decisions via the Polish HTA Agency (AOTMiT), whose guidelines were updated in 2016.

**Methods**: We identified key changes introduced by the update and, before guideline publication, analysed discrepancies between AOTMiT assessments and the submitting marketing authorisation holders (MAHs) to elucidate the context of the update. We compared the clarity and detail of the new guidelines versus those of the UK’s National Institute for Health and Care Excellence (NICE).

**Results**: The update specified more precise requirements for items such as indirect comparison or input data for economic modelling. Agency–MAH discrepancies relating to the subjects of the HTA update were found in 14.6% of published documents. The new Polish HTA guidelines were as clear and detailed as NICE’s on topics such as assessing quality of evidence and economic modelling, but were less informative when describing (for example) pairwise meta-analysis.

**Conclusions**: The Polish HTA guidelines update demonstrates lessons learned from internal and external experiences. The new guidelines adhere more closely to UK HTA standards, being clearer and more informative. While the update is expected to reduce Agency–MAH discrepancies, there remain areas for development, such as providing templates to aid HTA submissions.

## Introduction

Health technology assessment (HTA) is the systematic evaluation of the properties and effects of a health technology. The health technologies in question include medicines, devices, procedures and organisational systems that can be used to promote health, to prevent, diagnose or treat a disease, or for rehabilitation or long-term care [1]. HTA is a well-established instrument to support decision-making with regard to pricing and reimbursement (P&R) of health technologies in many countries around the world, including Poland.

The interest in harnessing health technology assessment (HTA) in Poland has been growing in the last decade, with major milestones being (1) the establishment of the national HTA Agency (*Agencja Oceny Technologii Medycznych i Taryfikacji*; AOTMiT) in 2005, which plays the role of an advisor to the Ministry of Health (MoH); (2) issuing the first HTA methodological guidelines in 2007 [2]; and (3) subsequent updates of these guidelines in 2009 [3] and 2016 [4]. Cooperation between AOTMiT and other European HTA societies, for example the European network for Health Technology Assessment (EUnetHTA), has also expedited the development of HTA and bolstered its use in Poland.

The HTA process in Poland has several specific stages, in which different bodies are involved. Briefly, the MoH is responsible for selection and prioritisation of topics, and accepts P&R submissions from marketing authorisation holders (MAHs), which include HTA reports. The HTA report is shared with AOTMIT for its own assessment (so-called ‘verification analysis’, which may challenge the HTA submission) and the subsequent appraisal process. The MoH may also commission the AOTMiT to reassess health technologies and services funded by the national health insurance system, to revise their coverage status.

Once the assessment by AOTMiT is complete, any interested stakeholder (mostly MAHs) can appeal the AOTMiT’s assessment by submitting comments within seven days, before a final recommendation is issued [5]. AOTMiT is obliged to address any submitted comments. The Agency’s output is the subject of appraisal by the Transparency Council.^1^ The Council prepares a statement to be considered as part of the final recommendation issued and published by the President of AOTMiT. There is a standardised format for the recommendation, which covers: a statement on public financing, rationale, objective of the recommendation, a brief note on the health problem, description of the technology and alternative technology/technologies, their efficacy, safety, relation of costs to health effects, impact on the payer’s budget and relevant recommendations from foreign HTA agencies, among others [6–8]. The entire process should take no longer than 60 days from the date when the Agency obtains an HTA report. The recommendation underpins the final decision of the MoH on the reimbursement of the health technology, although this is not binding.

Due to the development of the new Polish HTA methodological guidelines in 2016 [4], the objective of this research was twofold: (1) to identify its key updates versus the obsolete guidelines from 2009; and (2) to benchmark the new Polish guidelines against the UK’s National Institute for Health and Care Excellence (NICE) guidelines (2013 and 2015) [9,10] in terms of clarity and level of detail.

## Methodology

First, an analysis of key differences between the new (2016) and obsolete (2009) HTA methodological guidelines was carried out based on published, final documents. In addition to this task, a review of available, published AOTMiT’s assessments (verification analyses) and subsequent comments from MAHs during the appeal process from 2015 was performed, prior to the publication of the guideline update, to gain better understanding of the context for implementing the new HTA guidelines. AOTMiT’s verification analyses and subsequent comments submitted by MAHs during the appeal process were identified on the AOTMiT website [11]. An expected outcome of this additional task was the identification of potential subjects of differences between the assessments carried out by the Agency and those conducted by MAHs (henceforth ‘Agency–MAH discrepancies’) to understand whether they resonate with the topics of the guideline update.

A second research stream consisted of the analysis of similarities and differences between the new Polish guidelines and those from the English HTA body, NICE. The NICE guidelines considered in this research were: (1) ‘Guide to the methods of technology appraisal’, April 2013 [9], and; (2) ‘Single technology appraisal: User guide for company evidence submission template’, January 2015 [10]. Topic by topic, we looked at the clarity and level of detail necessary in a transparent and impartial appraisal process. The new Polish guidelines were broken down into 10 key topics: [1] Scope [2]; Evidence base [3]; Assessment of evidence quality [4]; Data synthesis: meta-analysis [5]; Data synthesis: indirect comparison [6]; Economic evaluation/perspective/time horizon [7]; Measuring and valuing health effects [8]; Evidence on resource use and costs [9]; Modelling; and [10], Uncertainty. All of these were analysed in the context of similarity to the NICE guidelines. Judgements on clarity and level of detail were made independently by two researchers, with a consensus reached in case of disagreements.

## Results

### Comparison of the new and obsolete Polish HTA methodological guidelines

Compared with the older version, the new Polish HTA methodological guidelines are more precise with respect to the different components of an HTA report. First, they recommend more detailed description of the health problem, including aetiology, pathogenesis, diagnosis, prognosis, complications, epidemiology, disease burden, therapeutic management and linking the disease to the ICD-10 classification (10th revision of the International Classification of Diseases). By comparison, the previous guidelines from 2009 required only basic information about the disease, including its natural history and prognosis as well as therapeutic and diagnostic management. Further, the new HTA guidelines also recommend description of the intervention of interest by providing its regulatory status, mechanism of action, Anatomical Therapeutic Chemical (ATC) classification code and link to the ICD-9-CM code (International classification system for surgical, diagnostic and therapeutic procedures), while the obsolete guidelines required merely a description of the marketing authorisation status and, if licensed, the date of approval in Poland or in other European Union (EU) member states (if not authorised in Poland). Third, the search strategy to identify information for a systematic literature review, as per the new guidelines, should be pursuant to the recommendations of the Cochrane Handbook [12] and the Centre for Reviews and Dissemination [13]. The guidelines from 2009, on the other hand, did not refer to these documents. The new guidelines also require to present the process for the selection of information using the PRISMA (Preferred Reporting Items for Systematic Reviews and Meta-Analyses) flow diagram [14,15]; previously, the QUOROM (Quality of Reporting of Meta-Analyses) flow chart [16] was recommended.

The new guidelines also propose a new approach to indirect comparison [4]. Namely, they advise using adjusted methods with control groups, such as Bucher’s method, Bayes method for mixed treatment comparison, Lumley’s network meta-analysis method, or meta-regression. Previous guidelines did not specify acceptable methods for indirect comparison. If it is impossible to carry out an indirect comparison without a control group, then the new guidelines recommend using methods with adjusted data (e.g., comparison with historical data, adjusting population characteristics). As to the quality assessment of studies included in HTA reports, AOTMiT recommends state-of-the-art instruments such as AMSTAR (a Measurement Tool to Assess Systematic Reviews and Meta-analysis scale for systematic reviews) [17], NICE scale for single-arm trials [18], or Cochrane’s Risk of Bias tool [12] for prospective trials with a control group. Previously, the Jadad scale [19] was recommended for experimental studies.

Moreover, new guidelines provide more specific criteria for the selection of clinical trial outcomes presented in the HTA report. In contrast, the guidelines from 2009 neither fully described nor extensively explained these criteria. For instance, the old guidelines did not require a specific description and justification of outcomes in the description of the health problem.

The new HTA guidelines provide more exhaustive and precise advice for economic analysis, particularly with respect to the modelling approach, cycle length, requirements for input data, and the need for external validation. These elaborate explanations were not provided in the 2009 guidelines. The perspective for the economic analysis became broader, with the new guidelines imposing a joint patient and payer perspective for technologies entailing co-payment, while in the 2009 guidelines such joint perspective was not envisioned.

As a result of AOTMiT’s involvement in EUnetHTA’s activities, the new Polish guidelines are now better aligned with EUnetHTA’s outputs [20–22]. Specifically, the new guidelines recommend using the HTA Core Model [23] for describing the health problem and current use of technology, including its technical characteristics, for assessing safety, clinical effectiveness as well as ethical, social, legal, and organisational impact. They also list additional databases which should be searched, such as the International Society of Pharmacoeconomics and Outcomes Research (ISPOR), Society for Medical Decision Making (SMDM), and Polish Pharmacoeconomics Society (*Polskie Towarzystwo Farmakoekonomiczne*; PTFE). These databases were not mentioned by the obsolete guidelines from 2009. Table 1 illustrates the main updates of Polish HTA methodological guidelines in a nutshell.

**Table 1.** Polish HTA guidelines 2016: what’s new since 2009.

More details on	Intervention (currently: regulatory status, mechanism of action, Anatomical Therapeutic Chemical Classification System (ATC) code, link to International classification system for surgical, diagnostic and therapeutic procedures (ICD-9-CM) code, among others; previously: unspecified characteristics and regulatory status)
	Health problem (currently: aetiology, pathogenesis, diagnosis, prognosis, complications, epidemiology, disease burden, therapeutic management, link to 10th Revision of International Classification of Diseases (ICD-10) code; previously: a description of the disease, its natural history, prognosis, therapeutic and diagnostic management)
	Search strategy (currently based on Cochrane Handbook, Centre for Reviews and Dissemination (CRD))
	Indirect comparison (currently methods are specified, including Bucher, Boyes, Lumley, meta-regression)
	Modelling approach with specifications regarding cycle length, input data, external validation, etc
Introduction of new tools to assess quality of systematic reviews and Randomised Clinical Trials (RCTs)	A Measurement Tool to Assess the Methodological Quality of Systematic Reviews (AMSTAR), Cochrane risk of Bias, NICE (single arm) standards as new tools for assessing quality of research
Revised assessment scope	Population, Intervention, Comparison, Outcome, Study (PICOS) instead of PICO
	More specific criteria for outcomes selection
New perspective in economic analysis	Joint patient and payer perspective for technologies entailing co-payment
Alignment with EUnetHTA	Use of the HTA Core Model for describing the health problem, the technology and its safety and clinical effectiveness, as well as ethical, social, legal and organisational impact
	Search strategy and selection of additional databases according to European network of HTA (EUnetHTA) guidelines

### Agency–MAH discrepancies

Eighty-seven assessments carried out by AOTMiT in 2015 were identified, with a total of 103 official documents listing the comments submitted by MAHs in response to the Agency’s assessment during the appeal process. Agency–MAH discrepancies that were related to the subjects of the 2016 HTA guidelines update were found in 15 (14.6%) of these official comment documents.

One of the most frequent issues which prompted AOTMiT to challenge the MAH’s HTA submission was related to the use of inappropriate input data for modelling. Another subject of disagreement between the Agency and MAHs concerned aspects of the search strategy and sources of information. The Agency argued that the search approach was too narrow, overlooking some important sources of information, such as Embase or the Cochrane Library. Ranking third as a source of discrepancy was the way in which the quality of trials and systematic reviews had been assessed; namely the Agency’s assessments differed from the ones performed by the MAHs [11].

Table 2 showcases the most frequent subjects of the Agency–MAH discrepancies that were related to the areas of the 2016 HTA guidelines update, ranked by the number of times they were mentioned in the official documents.

**Table 2.** Agency–MAH discrepancies in the perspective of topics of the HTA guideline update.

Subject of the HTA guideline update	Subject of Agency–MAH discrepancy (AOTMIT challenging the MAH’s HTA submission)	Total number of times mentioned in official documents
More details on modelling approach with regard to cycle length, input data and external validation	Inappropriate input data used for modelling (mentioned 11 times), e.g., modelling of drug consumption based on studies including the population that is only a part of the one defined in the assessment scope Cycle length (mentioned twice), e.g., modelling assumptions did not reflect the required (8- to 16-week) break between consecutive, annual treatment cycles	13
More details on search strategy and sources of information	Narrow search approach including the Medline medical database only Search not conducted in all the recommended sources of information, e.g., ISPOR Overlooking a research abstract that encapsulated relevant data	7
Introduction of new tools to assess quality of systematic reviews and RCTs (e.g., AMSTAR, Cochrane Risk of Bias)	Inappropriate assessment of the quality of a systematic review (by Cook criteria [24]) (mentioned three times) Inappropriate assessment of the internal validity of RCTs (by Jadad scale) (mentioned three times)	6
More details on indirect comparison (Bucher, Boyes, Lumley, meta-regression)	Objections regarding the appropriateness of methods for indirect comparison (e.g., unjustified use of a network analysis instead of Bucher’s method, or the use of regression or meta-regression with average baseline values instead of individual patient data)	4
New perspective in economic analysis (joint patient and payer perspective for technologies entailing co-payment)	Consideration of a payer perspective only instead of the joint perspective (e.g., failure to include additional patients’ medication costs identified by clinical experts)	1
**TOTAL NUMBER**		**31**

### Similarities and differences between the new Polish guidelines and NICE guidelines

Polish HTA guidelines (2016) [4] display many similarities with the NICE guidelines (2013, 2015) [9,10]. With regard to the scope, they provide ample and clear description of the PICOS scheme (population, intervention, comparator, outcomes, study design) and measures of health outcomes. Detailed and clear descriptions of the main and additional information sources, search strategy, selection process, and study characteristics are also rendered in detail. As evaluation of evidence quality is a key step in the HTA process, the Polish guidelines, similar to the NICE ones, set out exhaustive definitions of internal and external validity, and indicate proper tools for performing the validation, such as AMSTAR [17], Cochrane Risk of Bias [12], and NICE scale for single-arm trials [18]. Both guidelines require stating pre-defined inclusion and exclusion criteria, as well as quality assessment of included trials, with exhaustive explanations. For the indirect (mixed) comparison, the Polish guidelines provide a good level of detail on the specification of relevant methods, followed by indicating suggested reading, which corresponds closely to the English guides.

HTA guidelines from both agencies provide very similar and exhaustive recommendations on economic evaluation, including perspective and time horizon. Both guidelines list a range of acceptable types of economic evaluation, such as cost-effectiveness, cost-utility and cost-minimisation analyses. In addition, the two guidelines both define the acceptable time horizon as long enough to reflect all important differences in costs and outcomes that the technologies in question entail, and present descriptions of preferred measures, instruments and sources of utility data in similar detail. For modelling, there are clear recommendations for the components of a model, a checklist for critical appraisal and a summary of good practices in both guidelines; likewise, both contain an explanation of direct and indirect costs (followed by examples), and identification and accepted measures of resource use. To incorporate uncertainty analysis into the HTA process, the Polish and English guidelines provide explanation of the methods for deterministic and probabilistic sensitivity analysis, and outline the forms of results presentation (e.g., tornado plot, cost disutility plane, cost-effectiveness acceptability curve).

The analysis we conducted revealed that Polish HTA guidelines seem to touch upon some key components of an HTA report less comprehensively than the NICE guide. For example, the latter discusses measures of resource use and associated costs (e.g., staff, tests and monitoring costs), and provides pre-prepared sample templates for presenting a comparative summary of trial methodology, statistical analyses and study participants. This is not the case in the new Polish guidelines.

Differences between Polish and English guidelines continue with the provision of guidance on pairwise meta-analysis and key prerequisites for data synthesis, such as appropriate population, sample size and validity of evidence by the NICE guide. AOTMiT’s guidelines, in turn, describe the methods for meta–analysis only briefly and in a rather non-instructive manner, referring to external documents such as the Cochrane Handbook [12].

NICE in its guide considers the impact of adverse events on both health effects – measured by Health-related Quality of Life (HRQoL) decrement – and costs, transparently stating that this impact should be included in the cost-effectiveness analysis by accounting for the costs of managing adverse events associated with the technology in question. In the Polish guidelines, however, there is no clear recommendation for including the cost of adverse event management in the cost-effectiveness analysis. Adverse events are considered only in the context of the safety analysis. A summary of the comparison between the AOTMiT and NICE guidelines is presented in Table 3.

**Table 3.** Polish HTA guidelines (2016) versus NICE guidelines (2013, 2015).

	Similarity to NICE	Not addressed vs NICE
Scope	Ample and clear description of PICOS and measures of health outcomes	Measures of resource use/costs
Evidence base	Detailed and clear description of main and additional sources of information (clinical trial reports, papers, research) search strategy, selection process for relevant documents, clinical study characteristics	Detailed instruction on comparative summary of trial methodology, statistical analyses and study participants
Assessment of evidence quality	Explanation of internal/external validity and indication of proper tools (AMSTAR, Cochrane Risk of Bias, NICE standards)	Guidance on the Risk of Bias instrument and instruction for the assessment.
Data synthesis: meta-analysis	Explanation of pairwise meta-analysis, reporting key prerequisites for data synthesis (e.g., population, sample size, validity of evidence) assessment of heterogeneity. Polish HTA guidelines non-instructive, mostly referring to the Cochrane Handbook	
Data synthesis: indirect (mixed) comparison and NMA	Fair level of details on preferred methods with their specification, followed by suggested reading	Specification of search strategy. Study selection methods and outcomes of included studies
Economic evaluation/perspective/time horizon	Concise but exhaustive description that includes a range of possible types of economic evaluations (CEA, CUA, CMA), perspective (e.g., patients, carers and combined) and time horizon (long enough to reflect all important differences in costs or outcomes between the technologies being compared)	
Measuring and valuing health effects	Information on preferred measure (QALY), instrument (EQ-5D) and source of utility data	Information on the impact of adverse events on QALY estimate
Evidence on resource use and costs	Explanation of direct/indirect costs (followed by examples), identification and measures of resource use	Information on the impact of adverse events on resource use/costs
Modelling	Clear recommendations for the components of a model, the checklist for critical appraisal thereof and a summary of good practices	
Uncertainty	Explanation of methods for deterministic/probabilistic sensitivity analysis and forms of results presentation (tornado plot, cost disutility plane, CEAC)	Sources of uncertainty (choice of data sources)

AMSTAR – A Measurement Tool to Assess Systematic Reviews and Meta-analysis scale for systematic review; CEA – cost-effectiveness analysis; CEAC – cost-effectiveness acceptability curve; CMA – cost minimisation analysis; CUA – cost utility analysis; NMA – network meta-analysis; PICOS – population, intervention, comparison, outcome, study; QALY – quality-adjusted life year.

^1^The Transparency Council acts closely with the President of AOTMiT in providing independent advice/opinion on a health technology in question. Members of the Council are appointed by the Ministry of Health and consist of experts with clinical experience, representatives of MoH, National Health Fund, the Office for Registration of Medicinal Products, Medical Devices and Biocidal Products and the Commissioner for Patients’ Rights.

## Discussion

The main purpose of this study was to look closely into the nature of the changes introduced during the update of the Polish HTA guidelines, and to benchmark the new guidelines against the NICE guide. The NICE guide was chosen as the reference since, in England, a positive NICE recommendation is critical for securing National Health Service funding for the technology. Moreover, NICE guidelines are often referred to by other countries (including Poland) because of the perceived robust methodology of their review process.

The need for updating the HTA guidelines arose from the continuous evolution of HTA methods and other aspects of the process, and appears to have been inspired by both internal Polish HTA experiences and pan-European cooperation [4]. Since the last update of the HTA guidelines, numerous developments have taken place in Europe in terms of, for instance, efforts to harmonise the HTA process. EUnetHTA developed the HTA Core Model® in 2011 [23], which allowed more uniform HTA outputs across the different European countries. Indeed, the Model has become a prominent and comprehensive framework for assessing the value of health technologies. In our study, we highlighted that the new guidelines draw on this development by recommending the description of the ethical, social, legal, and organisational impact of heath technologies as proposed in the Model.

Our results showed a significant number of disagreements between the Agency and MAHs regarding the assessments performed in 2015. These disagreements matched the topics of the guideline update in 14.6% of official documents listing comments from MAHs. This illustrates that efforts made to clarify the potential topics of Agency–MAH disagreement, and to provide more detailed guidance, represent an approach that draws on internal experiences from the HTA process. We analysed only one year out of five that passed since the process of public consultations to AOTMiT’s verification analyses had been introduced, which may explain the somewhat small proportion of disagreements; however, the subject diversity of the identified disagreements led us to consider this sample fairly representative of the various types of issue encountered by MAHs during the HTA process. Although it is safe to say that some potential differences between AOTMiT and MAHs can be avoided thanks to the new, more precise guidelines, it is worth noting that disagreements may still appear, since their source lies in the complex nature of medical conditions and health technologies in question, and the associated difficulties with their assessment. For example, AOTMiT has challenged MAHs’ HTA submissions on grounds such as insufficient number of patients included in the groups of a randomised clinical trial (RCT). In another instance, the Agency challenged the evidence for an assessed technology due to the lack of RCTs. So far, there is no consensus on all the methodological aspects of HTA. In the aforementioned cases, even ideal HTA guidelines would not have prevented the Agency–MAH discrepancies from arising.

Our analysis demonstrated that the new Polish HTA guidelines are more in line with the UK HTA standards as represented here by the NICE guide, making a step towards the harmonisation of HTA processes and outputs in Europe – or at least in those countries where HTA is more economically driven (UK, Sweden), with clinical effectiveness as an important part of the assessment feeding the cost-effectiveness analysis. This could, for instance, mean that it will become easier for MAHs to prepare an HTA submission for a group of countries that share a similar approach to HTA. In Poland, the trend for drawing on external good HTA practices could mean that, in the future, AOTMiT’s recommendations may play a decisive role in the reimbursement of health technologies, similar to the recommendations of NICE in the UK. Nonetheless, recent years showed that positive AOTMiT recommendations were rather poorly associated with positive reimbursement decisions by MoH, agreeing in only 47%, 37% and merely 5% of cases in 2012, 2013 and 2014, respectively [25].

Kolasa et al. [26] performed a similar experiment to ours in 2012; but their analysis concerned the 2009 AOTMiT’s HTA guidelines, which they benchmarked against the Scottish Medicines Consortium (SMC) guidelines. In that study, the authors concluded that AOTMiT guidelines should be more precise, especially in the following areas: methodology for subgroup analysis, presentation of clinical evidence and methodology for the adaptation of economic models to the local setting. Authors pointed out that these improvements would result in a larger number of positive recommendations. Our analysis showed that the 2016 update of the AOTMiT guidelines indeed improved, especially in terms of clinical evidence presentation, and many other components not discussed by Kolasa et al., such as the methods for indirect comparison. Mathes et al. [27] performed a comparison of the economic evaluation methods used by the HTA agencies cooperating with EUnetHTA. Authors showed that the number, details and content of the guidelines vary strongly among the HTA agencies. These differences are caused by the disparate structure and regulation of the healthcare systems. The authors of that study concluded that there is a need for increased synergy between the guidelines issued by different HTA agencies. Our analysis showed that the recent update of the Polish HTA guidelines has made a step towards that goal, by improving consistency with the NICE guide in terms of clarity and level of detail. Harmonisation of HTA methods between agencies can facilitate the generalisability and transferability of HTA results between countries, as long as local data are used, and could therefore contribute to avoiding unnecessary duplication of work and the associated expenditures.

### Recommendations

Since HTA is an ever-evolving field in terms of the methods, processes and stakeholders, another update of the HTA guidelines is inevitable in years to come. AOTMiT may take example from the NICE guidelines and consider providing reporting templates and other tools to aid the submission of robust HTA reports in the future. Table 3 demonstrates not only the mismatches between the Polish and English HTA guidelines, but also encapsulates some recommendations for the next update of the HTA guidelines in Poland, with respect to the components that may need more comprehensive guidance, such as the impact of adverse events on health benefits and costs, or the sources of uncertainty in economic modelling.

### Study limitations

Our comparison of the new Polish HTA guidelines and the NICE guide might be biased by arbitrary judgment of what constitutes a ‘sufficient and clear’ recommendation. However, our approach involved two researchers making independent judgments, and reaching a consensus in case of disagreements, which mitigates the bias to some extent. Moreover, we looked at fairly objective measures, such as the provision of reporting templates by the NICE, which facilitates drafting an HTA report, or referral to external documents by the AOTMiT, which may impede straightforward understanding of a particular piece of recommendation. We made common-sense judgements on what meets the requirement for being comprehensive and providing clear guidance (e.g., the template provided by NICE) and what displays shortcomings (e.g., referral to external reading), although not every issue was black-and-white.

## Conclusions

AOTMiT has been striving for years to advance the HTA process, and the latest update of its HTA guidelines has contributed markedly to that progress. The update is an example of lessons learned from the appeal process. It is expected that there will be fewer discrepancies between AOTMiT’s assessment and MAH’s HTA report in the future, thanks to the more precise and detailed guidance on various areas of HTA such as modelling or indirect comparison. The new guidelines, with their more comprehensive approach, seem to adhere better to the NICE standards, through their improved clarity and level of detail, which is of pivotal importance for an impartial and transparent HTA appraisal, and gives rise to a more meaningful appeal process. Being more in line with HTA standards of countries where HTA plays a decisive role in the P&R process, the new HTA guidelines – as well as the international cooperation with EUnetHTA, coupled with a continuous internal learning process – are expected to result in an overall more transparent HTA process. In spite of the progress made with the recent HTA guideline update, there is still room for future improvements based on external HTA experiences, and, arguably, new needs that could emerge from the ever-evolving HTA process.
